# Detecting false intentions using unanticipated questions

**DOI:** 10.1371/journal.pone.0226257

**Published:** 2019-12-11

**Authors:** Glynis Bogaard, Joyce van der Mark, Ewout H. Meijer

**Affiliations:** Department of Clinical Psychological Science, Section Forensic Psychology, Maastricht University, Maastricht, The Netherlands; Arizona State University, UNITED STATES

## Abstract

The present study investigated whether measurable verbal differences occur when people vocalize their true and false intentions. To test potential differences, we used an experimental set-up where liars planned a criminal act (i.e., installing a virus on a network computer) and truth-tellers a non-criminal act (i.e., installing a new presentation program “SlideDog” on a network computer). Before they could carry out these acts, a confederate intercepted the participant and interviewed them about their intentions and the planning phase by using both anticipated and unanticipated questions. Liars used a cover story to mask their criminal intentions while truth-tellers told the entire truth. In contrast to our hypotheses, both human and automated coding did not show any evidence that liars and truth-tellers differed in plausibility or detailedness. Furthermore, results showed that asking unanticipated questions resulted in lengthier answers than anticipated questions. These results are in line with the mixed findings in the intention literature and suggest that plausibility and detailedness are less diagnostic cues for deception about intentions.

## Introduction

Most deception research has focused on detecting lies about past events. For example, research has investigated how existing lie detection tools can identify false autobiographical experiences [1–4], false alibis [5, 6] and false insurance claims [7, 8].

The terrorist attacks in the USA on 11 September 2001 marked the beginning of an era where terrorism dominates security agendas [9]. As a result, attention shifted from detecting lies about past events to detecting lies about intentions, because successfully detecting those lies would mean crimes could be prevented [10, 11]. Intentions are defined as an actor's mental state preceding his or her corresponding future actions [12]. They have three basic features that discern them from desires; they (1) relate solely to the intenders own actions, (2) come with strong commitment and (3) are based on some amount of planning [10]. In a legal context, especially false intentions, such as cover stories to conceal an actor’s real intention, are of particular relevance [13].

### Episodic future thought and intentions

Qualitative differences between true and false intentions can be predicted on the basis of episodic future thought (EFT; [14]). EFT explains our ability to mentally pre-experience a onetime personal event that may occur in the future and is a core component in the forming of true intentions [15]. To successfully accomplish EFT, we use details from the past–from our episodic memory–and use them to imagine future scenarios. This means that we can imagine an event we plan to act out, as long as we have some past experiences to rely on for simulating the future scenario. This process is very flexible and allows for the generation of an infinite number of hypothetical future scenarios [16, 17]. A related process that plays an important role for the formation of intentions is our ability to engage in ‘prospective memory’. When planning future events, we do not simply imagine them, we can actively predict and pre-experience the event, relying on past events to do so [18].

These processes form the basis for a possible discrimination between true and false intentions. A common approach for people with criminal intentions is to come up with a cover story, called false intention [19]. However, they lack the intent to execute this cover story. As a result, false intentions will lack the detailed representation—due to a lower level of engagement in EFT—that true intentions usually have. Therefore, reports about true intentions are expected to be more detailed than reports aimed at hiding criminal intentions [20].

### True and false intentions findings

A number of studies tested the difference between true and false intentions by interviewing participants about their travel plans. One of the first studies to investigate verbal differences between truth-tellers' and liars' intentions took place at an international airport in the United Kingdom [21]. Some passengers lied about the purpose of their trip but told the truth about the destination, while others told the entire truth. Liars made an equally detailed but less plausible statement than truth-tellers. These findings (i.e., difference in plausibility but not for details) were replicated with serving military and police officers [22]. Warmelink, Vrij, Mann and Granhag [23] showed in one experiment that true travel plans did include more spatial and temporal details than false travel plans. However, in a second experiment—using a more realistic setting—and in a direct replication [24], these differences did not emerge anymore. Two studies included automatic coding instead of human coding to investigate future travel plans. Kleinberg, Van Der Tool, Vrij and Arntz [25] instructed participants lie or tell the truth about their activities for the upcoming weekend. Contrary to their expectations, liars' statements were richer in location and person references than truth-tellers’ statements. Kleinberg, Warmelink, Arntz and Verschuere [26] investigated to what extent giving the instruction to describe their travel plans “as specific as possible” would magnify differences between true and false intentions. Although their manipulation successfully resulted in more information, no veracity effect for details was found.

In another line of research participants either plan and perform a mock criminal (i.e., false intention) or non-criminal (i.e., true intention) act but are intercepted before completing the planned act ([13]; but also see [11]). Furthermore, liars prepare a cover story in case they are intercepted. A major advantage is that the ground truth is known, and true and false statements are directly comparable as the activities and planning are experimentally controlled. Using this approach, Granhag and Knieps [20] found that true intentions lead to longer statements than false intentions. Sooniste et al. [27] found that truth-tellers used more words and details than liars when asked to report about how they planned the activity, but statements were equally long and detailed when asked about their intentions. Other studies also failed to find any differences in statement length and detailedness [28].

### Unexpected questions

One way to increase deception detection accuracy is to apply strategic interviewing [29, 30], such as asking unanticipated questions. I.e., the interviewer asks questions that an interviewee is unlikely to have prepared for. Such questions are designed so that truth-tellers can rely on their memory to simply recall an answer, while liars need to fabricate answers on the spot [27, 30]. The goal is to increase the cognitive demand for liars, but not for truth-tellers, resulting in more observable verbal differences between truth-tellers’ and liars’ statements. This approach has received empirical support for statements describing past events [27, 30–32]. For example, Lancaster et al. [31] reported that liars showed a decline in the reported visual and spatial details when moving from an anticipated to an unanticipated question.

Several studies investigated whether asking unanticipated questions would also amplify the verbal differences between true and false intentions. Warmelink et al. [33] showed that when asking expected questions, no verbal differences emerged between true and false intentions about travel plans, but liars did provide fewer details to unexpected questions than truth tellers. Similar findings were reported by Sooniste et al. [27] using a mock crime. Truth tellers and liars’ answers did not differ when asked expected questions, but verbal differences did occurr when answering unexpected questions. More recently, Sooniste et al. [34] investigated whether true and false intentions can be detected in small cells of suspects. Truth-tellers’ answers were more detailed compared with liars’ answers for both expected and unexpected questions. The authors also compared the consistency of the group members’ answers. For the expected questions, liars’ and truth-tellers’ answers did not differ, but liars’ reports were less consistent for unexpected questions. Additional support for the unanticipated question approach comes from research in aviation security screening [35]. This research showed that moving from predictable neutral questions to unanticipated questions changed the verbal behavior of liars (i.e., shorter and less informative answers) resulting in a better lie detection accuracy.

### The current study

Evidence for verbal differences between true and false intentions is mixed; while some studies report disparities in detail richness, others do not. Table 1 gives an overview of the difference between deceptive and true intentions in terms of details, plausibility and statement length. One potential explanation for the mixed nature of the findings can be found in the methodology of the studies. For example, researchers relied on different types of interviews (e.g., time prompts, model statement manipulation, time or route focus questions). It is not surprising that asking different questions may lead to different verbal cues. Moreover, some studies used human raters to code the statement, while others used computer coding. Although computer coding is more reliable than human coding [25], computers have a notoriously hard time taking context into account, potentially misclassifying words.

**Table 1:** Detailed overview of verbal differences between truth tellers’ and liars’ descriptions of intentions derived from published papers.

					Dependent variables		
#	Reference	Sample size	coding	Conditions	Details	Length	Plausibility
**Travel plans studies**							
1	Vrij, Granhag, Mann & Leal (2011)	60	H		-	-	< (m)
2	Vrij, Leal, Mann & Granhag (2011); Exp 1	62	H		-	/	< (l)
3	Warmelink, Vrij, Mann, Jundi & Granhag (2012)	86	H	WS: interview about general section	> (m)	/	/
				WS: interview about transportation section	< (m)	/	/
				WS: interview about core event	-	/	/
				WS: interview about planning	-	/	/
4	Warmelink, Vrij, Mann & Granhag (2013); Exp 1	86	H		< (s)	< (l)	/
5	Warmelink, Vrij, Mann & Granhag (2013); Exp 2	84	H	BS: interview without time prompt	-	< (l)	/
				BS: interview with time prompt	-	< (l)	/
6	Kleinberg, Nahari, Arntz & Verschuere (2017)	222	C	BS: information protocol	-	/	/
				BS: no information protocol	-	/	/
7	Kleinberg, van der Toolen, et. al. (2018); Exp 1	292	C		-	-	/
8	Kleinberg, van der Toolen, et. al. (2018); Exp 2	385	C	BS: interview without model statement	> (s)	-	/
				BS: interview with model statement	> (s)	-	/
9	Kleinberg, Warmelink, Arntz & Verschuere (2018)	79	C/H	WS: Interview with time focus	> (m)	-	/
				WS: Interview with route focus	> (m)	-	/
**Mock crime studies**							
10	Granhag & Kniep (2011)	70	H		/	< (m)	/
11	Sooniste, Granhag, Knieps & Vrij (2013)	70	H	WS: anticipated questions	-	-	/
				WS: unanticipated questions	< (l)	< (l)	/
12	Knieps, Granhag & Vrij (2013)	84	H	WS: immediate interview	-	-	/
				WS: delayed interview	-	-	/
13	Sooniste, Granhag, Strömwall & Vrij (2016)	232	H	BS: interview dyads	< (m)	/	/
				BS: interview quartets	< (m)	/	/

*Notes*. For interpretation of the significant effects we used the following symbols: “-” indicates no difference between truth tellers and liars, “>” indicates liars include this more while “<” indicates liars include this less, “/” means this variable was not tested. The effect size of the findings is provided between brackets: (s) = small effect, (m) = medium and (l) = large effect [36]. The variable “details” includes temporal, spatial and perceptual details. “H” refers to human coding of dependent variables, “C” means computer or automated coding. “BS” indicates a between subject manipulation and “WS” a within subject manipulation. For study #10 we did not include the detailedness rating because participants rated their own mental image of the intentions–not independent raters as in the other studies.

The current study aimed to replicate and extend previous research on intentions using the mock crime paradigm. Importantly, only four mock crime studies investigating intentions are currently published, of which three used the same manipulation [20, 27, 28]. I.e., liars were instructed to plan a mock crime consisting of placing a memory stick containing illegal material on a particular shelf in a shop in a mall, whereas truth tellers planned a non-criminal act: shopping for gifts in the mall. No background information was provided to the participants, e.g., they did not know why or for whom they had to do this. The fourth study [34] instructed liars to prepare a special lunch for which they had to get four items in the mall. Truth tellers were instructed to prepare a lunch for two exchange students and to gather the required goods from the local mall. Again, no further information was provided to the participants.

The current study used a mock crime and interception after which participants were interviewed using the unanticipated questions approach. We used a mock crime because of the benefit of knowing ground truth. In contrast to previous studies, we provided participants with more background information about their task. So, participants were informed why they were asked to perform the act. Furthermore, we explained that they could be intercepted. In sum, we investigated whether true and false intent statements differ in regard to detailedness (both human and automatic coding) and plausibility, and how the use of unanticipated questions would affect these variables. Based on previous findings, we expect verbal differences to be larger for unanticipated than for anticipated questions.

## Methods

### Participants

Based on the sample size in previous intentions studies using mock crimes, we included eighty participants (45 women; M = 22.92 years, SD = 4.26). The failure of three audio recordings resulted in 77 eligible participants. Participants were all undergraduates and were recruited by calls via an online participant pool, posters, flyers, social media, physical recruitment at lectures, the dining area and other places at the university and finally by use of the snowball method. The study was approved by the ethical committee of the Faculty of Psychology and Neuroscience of Maastricht University. Participants read and signed the appropriate informed consent in accordance with the Declaration of Helsinki and were guaranteed that they could resign from the project at any time and without any consequences. Datafiles for this study are available via https://hdl.handle.net/10411/JNAY8I.

### Procedure

#### Planning phase

Participants were told they were participating in a study on the security of the universities digital network ‘MAASnet'. They were informed there had been a substantial rise in viruses on university computers, posing a threat to network security, and that the IT-service desk had implemented a new protocol to minimize the risks of more viruses being installed. This policy would demand the service desk employees to conduct routine checks in the computer rooms to make sure no damaging content is uploaded on the computers. Participants were told that the current study examined the attentiveness of the employees. The explanation of this new policy implied that participants could be questioned by service desk employees if they were planning on uploading a program–damaging or not. Half of the participants (*N* = 38) had plan a mock-crime (i.e., lie intent), namely installing a virus program onto the network computer. The other half (*N* = 39) had to plan a non-criminal activity (i.e., true intent), namely uploading the presentation program SlideDog on a network computer. Participants were given 10 minutes to plan the activity, regardless of the condition. In the true intent condition, participants’ additional task was to use the SlideDog program to make a presentation about their ideal excursion (i.e., field or school trip for educational purposes) as a second part of the study. Participants in the false intent condition were instructed to fabricate a cover story to hide their criminal intentions in case they would get caught. For this cover story, they were encouraged to include the presentation program SlideDog and were given the same information as the truth tellers to use for their cover story. During the planning phase, all participants were provided with a USB-stick with the program that was to be uploaded onto the network computer, i.e. the SlideDog program for truth-tellers and a virus program for the liars. To motivate participants to not talk about the experiment, and stick to the cover story, they were told that they would not receive their participation credits if they told anyone that they were taking part in an experiment.

#### Interception

After the planning phase, the participants were asked to go to the computer room—and to keep the USB stick visible for observers—to prepare, and (supposedly) execute their planning. To make all participants believe that they were actually going to execute their planned actions they were intercepted *after* the participants entered the computer room. Specifically, participants were intercepted by a service desk employee (a confederate) once they had sat down behind the computer and started some preparation activities but had not yet inserted the USB-stick in the computer.

#### Interview

Interviews were conducted by two research assistants, each interviewed half of the participants. Before starting the study, they practiced the interview with each other and with the lead experimenter (first author). To make sure the interviewers would not deviate from the predetermined questions and the order, they used a script (see below).

The interviewer took the participant to a private room where s/he was told that there were numerous viruses on computers of the ‘MAASnet' and according to a new protocol everyone who was working in the computer room was questioned in order to warrant the safety of the university network. They were told that if they gave the interviewer unsatisfying answers or in any other way raised suspicion, he would call his manager for further questioning. To culminate high-stakes, and motivate the liars, it was also mentioned that this manager could hold the participant liable for the possible costs resulting from a virus on the computer. The interviewer asked the following questions, and the interview was audio recorded.

1. ‘Can you tell me what you intended to do in the computer room?’
2. ‘What did you intend to do with your USB-stick?’
3. ‘What is your presentation about?’
4. ‘What is the presentation for?’
5. ‘Can you tell me more details about the presentation you intended to make’

The interviewer continued with five questions, which were directed towards the planning of the intended activities. Previous intention research has shown that planning questions are considered to be more unanticipated than questions about the actual intention [19, 27, 34], and questions about the goal of the act [33].

6 ‘Which information sources did you plan to use for your presentation?’
7 ‘Which part of your presentation did you plan to emphasize?’
8 ‘Can you tell me how you planned the outline of your presentation to be?’
9 ‘Which part of the presentation do you find hardest to plan?’
10 ‘Which part of your presentation do you find least difficult to plan?’

Following Warmelink et al. [33], we started with questions about their intentions, and then moved to questions about the planning phase at a later stage [27, 33, 34]. The rationale they provided for this order is that when participants prepare for an interview, they tend to only prepare the expected questions (see [33] for more information). After they had answered the questions, participants were told that the interviewer was a confederate in the research. Before debriefing, the participants were asked to fill in a post-interview questionnaire on a computer.

#### Post-interview questionnaire

After the participant had given her or his statement s/he was informed about the goal of the study and provided with a post-interview questionnaire. Importantly, for participants in the false intent condition, this questionnaire started with a separate section making it clear that the role-playing part of the study was now over, and all questions should be answered truthfully. Participants were asked to answer the following six questions on a 7-point Liker-scale: (1) To which extent did you lie during the experiment? (2) To which extent did you believe you would be held liable for the costs? (3) To which extent did you believe the cover story, i.e. testing new protocol for ICT-staff? (4) How nervous did you feel during the experiment? (5) To which extent did you have trouble with planning? (6) To which extent are you satisfied with your preparations?

Next, participants were presented with the ten interview questions again, and asked whether each question was unexpected (yes or no) or hard to answer (yes or no).

### Coding and reliability

Detailedness and plausibility were coded by two raters who were both blind to the veracity status of the statements, and the answers to the anticipated and unanticipated questions were coded separately. The following variables were coded: (1) the number of spatial details, (2) the number of temporal details, (3) the number of perceptual details and (4) how plausible the answer was on a Likert scale ranging from 1 = implausible to 5 = plausible. To establish coder reliability, the first coder coded all the statements, while the second rater coded the answers of 20 participants that were randomly drawn from the dataset. Inter‐rater reliability for the frequency variables was calculated by means of an intraclass correlation coefficient (ICC). Values for the statements varied between 0.66 and 0.98, with an average of 0.83. These results are typical in the field (e.g., 23, 24, 27) and indicate a moderate‐to‐high agreement and are sufficient to continue subsequent analyses (see [37]).

The length of the responses was objectively coded via the Linguistic Inquiry and Word Count (LIWC; [38]). LIWC is a software program that analyses written statements word by word and stores them into several word categories. The number of words in each category is counted, adjusted for the number of total words used, and is expressed as frequency per 100 words [38, 39]. We used the Dutch translation of the 2007 LIWC version [40]. To prepare the transcripts for analysis, all utterances from the interviewer and filler words (e.g., err, uhm) were excluded. As for the human coders, answers to the anticipated and unanticipated questions were coded separately.

Like previous studies, we modeled detail richness by using the following LIWC categories “senses” (e.g., appear, speak), “space” (e.g., wide, under), and “time” (e.g., during, until) [24–26]. These categories respectively represent the perceptual, spatial and temporal details included in the statements. To compare our results with previous intentions studies, we summed the three categories “senses”, “space” and “time” for all participants to create the variable “detail richness”.

### Design

A 2 (Veracity: truthful intent vs. false intent) by 2 (Question Type: anticipated vs. unanticipated) split plot design was employed, with veracity as a between-subject factor, and question type as a within factor.

## Results

### Pre-analysis check

To check whether our participants complied with the instructions to lie or to tell the truth we conducted several one-way ANOVAs. We also checked whether there were any differences between truth-tellers and liars with regards to which extent they believed our cover story, they believed they would be held liable for the costs, and the extent to which they were nervous during the interview. Lastly, we checked the effort participants put in their planning.

Results revealed that the participants indeed followed the instructions; participants in the false intent condition told significantly more lies during the interview (*M =* 2.00, *SD =* 1.16) than participants in the truthful intent condition (*M =* 4.87, *SD =* 1.92), *F* (75) = 62.54, *p* < .001, *95% CI* [1.62,2.38],[4.25,5.49], *d* = 1.83. The degree to which participants believed the cover story did not differ significantly between liars (*M* = 4.08, *SD* = 1.63) and truth-tellers (*M* = 4.33, *SD* = 2.08), *F* (75) = 1.14, *p* = .55, *95% CI* [3.54,4.62], [3.66,5.01], *d* = .08. Furthermore, liars (*M* = 2.87, *SD* = 1.63) and truth-tellers (*M* = 2.74, *SD* = 1.88) did not differ in in how much they believed they would be held liable for the costs, *F* (75) = .10, *p* = .75, *95% CI* [2.33,3.40], [2.13,3.36], *d* = .14. Lastly, there was no significant difference in the level of nervousness for liars (*M* = 3.82, *SD* = 1.60) and truth-tellers (*M* = 3.74, *SD* = 1.77), *F* (75) = .04, *p* = .85, *95% CI* [3.29,4.34],[3.17,4.32], *d* = .05.

Participants had to rate their perception of the planning and the extent to which they were satisfied with their planning. Participants in the truthful intent condition (*M =* 2.79, *SD* = 1.51) found it equally difficult to plan their activities as participants in the false intent condition (*M =* 2.89, *SD =* 1.56), *F* (75) = .08, *p* = .78, *95% CI* [2.31,3.28],[2.38,3.41], *d* = .07. The extent to which the participants were satisfied with their planning also did not significantly differ between both groups (truthful intent: *M =* 4.90, *SD =* .1.70, false intent: *M =* 4.87, *SD =* .1.83, *F* (75) = .005, *p* = .94, *95% CI* [4.35,5.45],[4.27,5.47], *d* = .02).

### The anticipation and difficulty of questions–manipulation check

To investigate anticipation and difficulty, we recoded the answers to the post-interview questionnaire about the anticipation and difficulty in the following way: “yes” answers were recoded as 1 and “no” as 0. Next, we computed a sum score for the five intention questions and the five planning questions and compared these scores. As expected, a paired-samples t-test revealed that participants perceived the questions about the planning (*M =* 2.83, *SD =* 1.61) as significantly less anticipated than questions on the intentions (*M =* 1.19, *SD =* 1.29), *t* (76) = -7.33, *p* < .001, *95% CI*_*diff*_ [-2.08,-1.19], *d* = 1.13. Also, questions about the planning (*M =* 1.83, *SD =* 1.20) were perceived as significantly more difficult than questions about the intentions (*M =* 0.80, *SD =* 0.73), *t* (76) = -6.34, *p* < .001, *95% CI*_*diff*_ [-1.37,-0.71], *d* = 1.04. Based on these results, planning questions were considered unanticipated, while intention questions were considered anticipated.

### Details and Plausibility

Parts of two recordings were inaudible, and therefore could not be properly transcribed. These two participants were removed, resulting in a total of 75 participants. Six 2 (veracity) x 2 (anticipation) repeated measures ANOVA with veracity as a between-subject factor and anticipation as a within-subject factor were conducted, one for each dependent variable (length, plausibility, spatial details, temporal details, perceptual details, and all details combined). To correct for multiple testing, we used an alpha of .008 (.05/6).

#### Total number of details

Results revealed a main effect for anticipation as participants included more details in their answers to the unanticipated questions than the anticipated questions [*F* (1, 73) = 16.01, *p* < .001, *n*_*p*_^*2*^ = .18]. There was no main effect for veracity [*F* (1, 73) = 4.06, *p* = .05, *n*_*p*_^*2*^ = .05] and no significant interaction effect [*F* (1, 73) = .14, *p* = .71, *n*_*p*_^*2*^ < .01]. See Table 2 for means and standard deviations.

**Table 2:** Means and standard deviations for details and plausibility.

	Truth-tellers					
	Anticipated			Unanticipated		
	M	SD	95% CI	M	SD	95% CI
Total details	3.05	3.73	1.77,4.34	5.16	4.41	3.6,6.70
Spatial details	1.89	2.76	1.13,2.66	1.53	1.75	0.76,2.29
Temporal details	1.08	1.40	0.43,1.73	3.34	3.56	2.26,4.43
Perceptual details	0.08	0.27	-0.15,0.30	0.29	0.65	0.11,0.47
Plausibility	3.55	0.98	3.21,3.90	3.58	1.06	3.25,3.91
	Liars					
	Anticipated			Unanticipated		
	M	SD	95% CI	M	SD	95% CI
Total details	4.51	4.22	3.21,5.82	7.05	5.12	5.49,8.62
Spatial details	2.16	1.89	1.39,2.94	2.46	2.86	1.68,3.24
Temporal details	2.00	2.51	1.34,2.66	4.41	3.12	3.31,5.50
Perceptual details	0.35	0.95	0.12,0.58	0.19	0.46	0.01,0.38
Plausibility	3.59	1.14	3.25,3.94	3.92	0.95	3.59,4.25

#### Spatial details

Results showed no main effect of veracity [*F* (1, 73) = 1.96, *p* = .17, *n*_*p*_^*2*^ = .03] or anticipation [*F* (1, 73) = .01, *p* = .92, *n*_*p*_^*2*^ < .01] and no significant interaction effect [*F* (1, 73) = .96, *p* = .33, *n*_*p*_^*2*^ = .01].

#### Temporal details

Results did show a main effect for temporal details [*F* (1, 73) = 35.08, *p* < .001, *n*_*p*_^*2*^ = .33]; participants included more temporal details when answering unanticipated than anticipated questions. There was no main effect for veracity [*F* (1, 73) = 3.89, *p* = .05, *n*_*p*_^*2*^ = .05], and no interaction effect [*F* (1, 73) = .03, *p* = .86, *n*_*p*_^*2*^ < .01].

#### Perceptual details

There were no significant differences in perceptual details, no main effect of anticipation [*F* (1, 73) = .06, *p* = .81, *n*_*p*_^*2*^ < .01] or veracity [*F* (1, 73) = .68, *p* = .41, *n*_*p*_^*2*^ = .01] and no interaction effect [*F* (1, 73) = 3.32, *p* = .07, *n*_*p*_^*2*^ = .04].

#### Plausibility

There were also no significant differences in the plausibility of our participants’ answers. Anticipated and unanticipated questions resulted in equally plausible answers [*F* (1, 73) = 1.30, *p* = .26, *n*_*p*_^*2*^ = .02], truth-tellers’ and liars’ answers were also equally plausibility [*F* (1, 73) = 1.09, *p* = .30, *n*_*p*_^*2*^ = .02], and there was no interaction effect [*F* (1, 73) = .94, *p* = .34, *n*_*p*_^*2*^ = .01].

### LIWC coding

We checked whether true and false intention statements differed in detail richness coded using LIWC. Four 2 (veracity) x 2 (anticipation) repeated measures ANOVA with veracity as a between-subject factor and anticipation as a within-subject factor were conducted, one for each dependent variable (spatial details, temporal details, perceptual details, and all details combined). To correct for multiple testing, we used an alpha of .013 (.05/4).

#### Total detail richness

Answers to anticipated questions did not differ in detail richness from unanticipated questions, indicating there was no significant main effect of anticipation [*F* (1, 73) = .12, *p* = .73, *n*_*p*_^*2*^ = .002] and no significant interaction effect [*F* (1, 73) = 2.63, *p* = .11, *n*_*p*_^*2*^ = .04]. As with human coding, due to our alpha correction, there was also no main effect of veracity [*F* (1, 73) = 6.21, *p* = .015, *n*_*p*_^*2*^ = .08]. See Table 3 for means and standard deviations.

**Table 3:** Means and standard deviations for LIWC coded details.

	Truth-tellers					
	Anticipated			Unanticipated		
	M	SD	95% CI	M	SD	95% CI
Total details	6.40	2.88	5.46,7.34	6.97	3.26	6.03,7.90
Spatial details	2.60	2.40	1.93,3.27	1.98	1.70	1.48,2.49
Temporal details	2.55	1.55	1.92,3.19	3.68	2.61	2.98,4.38
Perceptual details	1.25	1.28	0.85,1.64	1.31	1.01	0.96,1.66
	Liars					
	Anticipated			Unanticipated		
	M	SD	95% CI	M	SD	95% CI
Total details	8.37	2.29	7.42,9.32	7.49	2.46	6.54,8.44
Spatial details	3.00	1.65	2.33,3.68	2.19	1.38	1.68,2.70
Temporal details	4.07	2.32	3.42,4.71	4.13	1.58	3.42,4.84
Perceptual details	1.30	1.16	0.90,1.70	1.17	1.15	0.82,1.53

#### Spatial details

Results showed no main effect of veracity [*F* (1, 73) = .78, *p* = .38, *n*_*p*_^*2*^ = .01] and no significant interaction effect [*F* (1, 73) = .16, *p* = .69, *n*_*p*_^*2*^ = .01]. Results did show a main effect for anticipation [*F* (1, 73) = 8.58, *p* = .005, *n*_*p*_^*2*^ = .11] because anticipated questions resulted in more spatial details than unanticipated questions.

#### Temporal details

Results showed a main effect for veracity [*F* (1, 73) = 8.57, *p* = .005, *n*_*p*_^*2*^ = .11]; liars included more temporal details than truth tellers. There was no main effect for anticipation [*F* (1, 73) = 3.01, *p* = .09, *n*_*p*_^*2*^ = .04], and no interaction effect [*F* (1, 73) = 2.43, *p* = .12, *n*_*p*_^*2*^ = .03].

#### Perceptual details

Lastly, there were no significant differences in LIWC coded perceptual details, no main effect of veracity [*F* (1, 73) = .04, *p* = .84, *n*_*p*_^*2*^ < .01] or anticipation [*F* (1, 73) = .04, *p* = .85, *n*_*p*_^*2*^ < .01] and no interaction effect [*F* (1, 73) = .27, *p* = .61, *n*_*p*_^*2*^ < .01].

### Statement length

We checked whether true and false intention statements differed in word count. There was a significant main effect of anticipation [*F* (1, 73) = 41.53, *p* < .001, *95% CI* [86.37,116.01],[134.81,173.03], *n*_*p*_^*2*^ = .36]; answers to anticipated questions (*M* = 101.12, *SD* = 64.17) were shorter than to unanticipated questions. False statements (*M* = 153.83, *SD* = 82.73) did not significantly differ from true statements (*M* = 121.64, *SD* = 76.52) [*F* (1, 73) = .61, *p* = .44, *95% CI* [100.53,142.76],[112.06,154.86], *n*_*p*_^*2*^ = .008] and there was no significant interaction effect [*F* (1, 73) = .03, *p* = .86, *n*_*p*_^*2*^ < .01].

## Discussion

The current study investigated whether true and false intent statements differ in verbal cues and whether these differences can be magnified using unanticipated questions. We did not find any evidence that liars were less detailed than truth-tellers. On the contrary, numerically, liars included more details–both for human and automated coding—but after our correction for multiple testing this was no longer significant. With the automated coding, this effect was predominantly driven by liars including more temporal details than truth tellers. For the human coding, results showed that asking unanticipated questions resulted in more details than anticipated questions and this effect was driven by temporal details only. On the other hand, automated coding showed anticipated questions resulted in more spatial details than unanticipated questions. Lastly, unexpected questions resulted in lengthier answers than expected questions.

Liars and truth-tellers did not differ with regards to overall detail richness. In line with these findings, some previous studies failed to show any verbal differences [21, 22, 26, 28, 33]. In contrast, other studies reported that true intentions included more temporal and spatial details than false ones [20, 23]. There is even evidence that liars sometimes include more details than truth-tellers [25], a finding that was supported by our automatic coding results. It is important to note that overall, people provide fewer details when describing intentions than past activities [22], most likely because participants are missing the experience of the actual event to draw on. This could make it easier for liars to mimic the level of detailedness of truth-tellers in their statement, explaining the mixed findings.

Contrary to our results, previous studies reported that the use of unexpected questions magnified verbal differences between true and false intent statements [27, 30, 33, 41]. For example, Sooniste et al. [27, 34] showed that truth-tellers’ answers to planning questions were lengthier and more detailed than liars’ answers. Our automatic coding results were partially in line with these findings because unexpected questions resulted in less spatial details than expected questions. Yet, human coding showed that unexpected questions resulted in more temporal details than expected questions. Furthermore, overall, unexpected questions resulted in lengthier answers than expected questions. A finding that has also been reported by Kleinberg et al. [26]. They explain their counterintuitive findings might have occurred because unexpected questions–although rated less anticipated—were perhaps not harder to answer than expected questions. However, given the current study successfully manipulated both factors, difficulty does not seem to be a fitting explanation. Another possibility explaining why participants gave more elaborate answers to unexpected questions is an order effect. As in previous studies, participants were always asked expected questions first, followed by unexpected questions. Perhaps, participants felt more at ease as the interview progressed which resulted in more information and more details overall for later questions, regardless of the accounts’ veracity status.

It is also possible that planning questions are not the best type of questions to magnify verbal differences for true and false intentions. For example, Parkhouse and Ormerod [42] tested several types of unanticipated questions and showed that planning questions were least likely to result in verbal differences between truth tellers and liars when compared with spatial and temporal questions. However, given our study investigated intentions—meaning an event that did not take place yet—truth tellers and liars would not know the answer to these types of questions. Perhaps studies should look into other types of questions that can be used to better identify false intentions.

Although both automatic and human coding showed no verbal differences between true and false intentions, the two coding styles did differ with regards to the type of questions asked. Differences between human and automatic coding can be expected for two reasons. First, although automatic coding is more reliable [25], human coders can take context into account, which leads to a more flexible coding system. Second, LIWC reports proportion scores, thus LIWC scores are corrected for the length of the statement [38]. Previous studies have shown that after correcting for the length of the statements, results can change considerably [43, 44].

Two explanations for our null findings can be given. First, our lack of finding verbal differences between true and false intentions dovetail nicely with the recent paper by Luke [45] who, based on multiple simulations, concluded that the scientific lie detection literature offers very weak evidence for the existence of observable differences between true and deceptive accounts. Although detail richness was shown to be the most reliable indicator of deception about past event, this effect may not translate to deception about future events. Importantly, Luke (45] showed that the effect sizes of many deception cues may be greatly overestimated. Several reasons can account for these inflations; for example, publications bias, small numbers of estimates and low power. None of the reviewed studies on intentions reported a priori G*power analysis ensuring sufficient power–including the current study. Nonetheless, our study with 80 participants is comparable to previous intentions studies that have been published using a similar manipulation (varying between 60 and 86 participants). In all, these findings show that great care should be taken when making recommendation about the use of verbal cues between true and false intentions.

A second potential explanation is more modest and is that verbal differences did not emerge in our task. It is easily imaginable that task difficulty and topic familiarity play a moderating role in the extent to which people are able to pre-experience their intentions. Based on the information the participants told the research assistants, some had difficulties with preparing the excursion as it was an unfamiliar topic to them. It might be that the veracity status (i.e., true vs. false intent) is less important for the level of detailedness in the provided statements than the familiarity of the intention described. Evidence for this explanation was provided by Warmelink et al. [33], who showed previous experience with a forthcoming trip was associated with more detail richness in true and false intentions. Other studies have also shown that location familiarity and the amount of contextual knowledge influence how successful people are in engaging in EFT [46, 47].

Our results showed that some participants in the truthful condition also told lies, while participants in the deceptive condition also told some truths. One could wonder whether this can explain our lack of significant results. But for this variance to explain the difference in findings between our study and others, one would need to assume that in previous studies, participants in the truth condition were substantially more truthful. This may be unlikely, as other recent research shows it is not rare for liars to include truths [48–50], and truthtellers to include lies [51–54].

Our results have practical implications. Although detail richness has found strong support as a cue to detect deception in accounts of past events [4, 45, 55, 56], its usefulness for discriminating between true and false intentions is unclear. Our findings indicate that this strategy might be ineffective, especially for immigration officers questioning travelers about the purpose of their trips–even when asking unexpected questions. Using "lack of details" as a decision rule might even lead to a wrongful decision as some studies indicated false intentions sometimes include more details than true intentions [24, 25, 33].

Reviewing the existing scientific literature shows that our null findings are not isolated; multiple experiments, questioning approaches, manipulations and designs reported similar results. In conclusion, verbal differences between true and false intentions seem to be faint and changeable.
